# Portable Colorimetric Device with Commercial Microplates for Quantitative Detection of Urine Biomarkers: Design, Development, and Clinical Evaluation

**DOI:** 10.3390/bios12090723

**Published:** 2022-09-04

**Authors:** Anh Tran Tam Pham, Damian Tohl, Qi Hu, Jordan Li, Karen J. Reynolds, Youhong Tang

**Affiliations:** 1 Australia-China Joint Research Centre for Personal Health Technologies, Medical Device Research Institute, Flinders University, Tonsley, SA 5042, Australia; 2 Department of Renal Medicine, Flinders Medical Centre, College of Medicine and Public Health, Flinders University, Bedford Park, SA 5042, Australia

**Keywords:** colorimetric sensing, urine biomarkers, portable medical device, clinical evaluation, rural areas

## Abstract

Urine biomarkers are important in monitoring diseases related to human kidney function. The current processes for measuring biomarker levels in urine samples require patients to regularly visit clinical facilities, which is inconvenient and sometimes impossible for patients in rural areas. Therefore, portable analysis devices for the measurement of urine biomarkers are urgently requested. In this study, a portable platform using colorimetry, a common and simple-to-operate chemical analysis technique, was developed to measure urine biomarkers. The device, using commercial test kits as recognising reagents and a 96-well microplate as a solution container, provides quantitative measures of biomarker concentration. Moreover, the proposed device introduces a calibration method to minimise the dependence of regular maintenance. The device’s performance was evaluated with urine from 73 renal patients and its results matched with clinical results well. The device has the potential for measuring urine creatinine, in addition to performing a variety of commercial assays for biomarker detection in human body fluids in general.

## 1. Introduction

Colorimetric examination is a common analytical method for measuring chemicals in both research and clinical fields. The principle of this method is to observe the colour changes of a mixture solution of the specific analyte and the recognising agent due to their chemical or biochemical reactions [1,2,3,4,5]. The operator can read the quantitative result of the chemical presence by observing the colour changes of the mixture with the naked eye [1,3,4,6,7]. Thanks to the rapid results, friendly operation, and simple storage, the colorimetric detection method has been used widely in society, such as the haemoglobin blood test, glucose test strip, and urine test strip, or commercial test kits for solution-based tests. Of these tools, commercial test kits are being used for research and in clinical laboratories due to their high sensitivity, various analyte tests, and especially, quantitative results. The effective accessory supporting commercial test kits is the microplate, which can be described as a pattern of microwells, equipped on a flat platform. The microplate supports the measurements of multiple samples at the same time, saving time and effort for the users. There are several types of microplates with various numbers of wells, i.e., 6, 24, 48, 96, or 384 wells, and different colours, i.e., black, white, or clear [8,9]. Currently, the 96-well microplate (96-WM) is the plate most used in research and clinical facilities as it provides more options for the number of tests and has standard dimensions for use with commercial testing instruments [10,11]. Although they can support multiple tests simultaneously, 96-WM reading requires specific equipment to examine multiple wells in the plate, which is usually cumbersome and non-portable, and this causes the operators to remain in a laboratory environment [12,13,14]. In recent years, researchers have focused on developing portable devices, which can examine the microplate reading to support the measurement in the remote testing conditions. In 2016, Feng et al. developed a portable 3D-printed device using a smartphone to perform the antimicrobial susceptibility testing (AST) on a 96-well microplate, targeting the gram-negative bacteria, *Klebsiella pneumoniae* with the measurement accuracy being over 95% [11]. In the same year, Fu et al. introduced another smartphone-based device, using commercial chemicals to measure various biomarkers spiked in blood and urine samples [12]. Their device showed good results in the limit of detection of 17.54 U/L for alanine aminotransferase, 15.56 U/L for alkaline phosphatase, 0.00135 mmol/L (1.35 μM) for creatinine spiked in urine samples, and other biomarkers. Other researchers also reported smartphone-related devices developed using microplate with clinical-approved testing kits to detect particular biomarkers of infectious diseases, such as varicella zoster virus IgG (VZV) for detecting chicken pox (varicella) and shingles (herpes zoster), cytomegalovirus IgG (CMV) for detecting herpes viruses, or the protein CFP-10 for detecting tuberculosis [13,15], female reproductive steroid hormone profiles [14], or the progesterone concentration in the whole blood sample [16]. Although these devices reported high sensitivity and stability, most are only used in research applications. In these devices, a smartphone is commonly used as the optical sensor, but the rapid development of mobile technology and the varied dimensions of smartphones create compatibility issues, and the internal processing of the optical cameras affect measurement accuracy [5]. Furthermore, smartphone use introduces issues with user confidentiality and cross-contamination hazards [5,17]. 

Another parameter for consideration when developing a chemical analysis device is the target analyte. As mentioned above, portable colorimetry devices can detect various chemical or biomolecules in solutions, which can be used as biomarkers, such as the presence of albumin in urine indicating kidney disease, a reduced citrate level in urine suggesting prostate cancer, the level of cardiac Troponin I (cTnI) in blood helping the diagnosis of acute myocardial infarction, or the absence of glutathione in saliva indicating the potential of head-neck cancer [5,18,19,20,21]. The detection target will influence the design choices, such as the sensitivity of the detecting sensor, the background light intensity, and the recognising reagent type. In this research, urine has been chosen as the target biofluid due to its clinical importance in healthcare and the non-invasive method of collection. The chosen biomarkers are creatinine and glucose, which are two of the common biomarkers tested in human urine examination. In normal conditions, creatinine in urine varies from 2.5 to 17 mmol/L (28.28 to 192.304 mg/dL), while glucose is not present in urine [22]. The presence of glucose in urine is a sign of biological dysfunction, whilst a high concentration of creatinine may be an indication of diabetes, muscle damage, or kidney disorder [23,24]. In clinical urine tests, creatinine and glucose in urine samples can be first observed by using dip sticks. However, the dip-stick method only provides initial screening; therefore, medical staff often measure them on automated analysers using colorimetric or enzymatic assays with high accuracy and reliability. If the measurements from these methods can be equipped into a compact portable device, it will provide potential for use in rural and remote areas.

In this report, we have developed a portable colorimetry device to facilitate a 96-WM with a commercial test reagent. The targets of detection biomarkers in this research are glucose and creatinine, two common biomarkers present in human urine. The device was tested with two commercial test reagents, and its performance compared firstly with results from a clinical microplate reader, and secondly, using urine samples from renal patients and comparing the results with clinical results provided by a local pathology laboratory.

## 2. Device Design, Working Mechanism and Post-Processing Algorithm 

### 2.1. Device Design and Fabrication

Following the principle of a colorimetric reader, the device is comprised of four main components: a camera, a microplate, a white light source with detachable LEDs, and a controlled optical environment for the colorimetric measurement. During design and fabrication, there were several features to be considered to support device performance, such as a uniform background luminance for the measurement, a suitable luminance source to improve the quality of the optical measurement, an optical sensor to capture the visible colour change of the chemical, a modifiable structure to support the requirements of different measurements, and dimension and weight to support the device’s portability. 

The device was designed and developed to have the flexibility to work with multiple testing chemicals and optical wavelength requirements. The device hardware provides a controllable optical environment for the measurement. A detachable LED light source provides the flexibility to change LEDs for different chemical test requirements, where certain testing chemicals show a higher sensitivity with a certain optical wavelength. Moreover, 3D printing technology was used to achieve lightweight, affordable, and simple fabrication. A low-cost Raspberry Pi camera (Raspberry Pi Australia) with wide visible light spectrum sensitivity was used to capture the colour changes of the chemical mixtures. Image processing was applied for device calibration and colour change detection to increase the accuracy and robustness of the measurements. The device has the capacity to test multiple biomarkers with minimal operation. The device also has the potential to be extended for testing with other technologies, such as paper test strips, which have been employed in portable medical device development for many years [25,26]. The detailed design of the device is explored below.

#### 2.1.1. The Camera and Microplate

**Figure 1:**
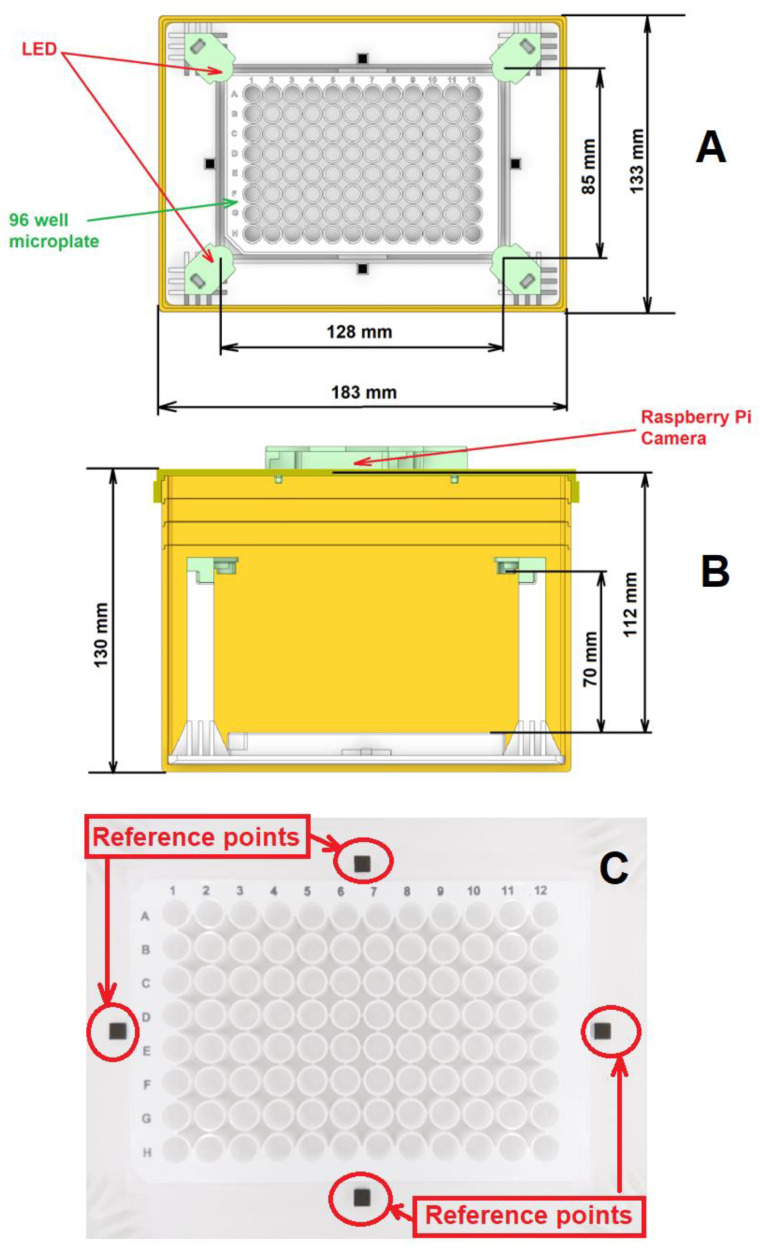
(**A**,**B**) The sketch of a 96-well microplate colorimetry prototype; and (**C**) a white 96-well microplate image taken by a Raspberry Pi camera in the proposed device (Dark squares are four reference points for image process).

There are different types of 96-WM, specifically transparent and opaque plates. The transparent microplate is a common choice for the bottom-reading measurement performed by microscopy, which is often operated in laboratory-condition spaces, whilst opaque microplates, white or black, are used for top-reading measurements. In this device, the colour solution is observed from the top and the colour development is captured by a digital camera; thus, a white opaque 96-WM was employed for the device. The 96-WM has dimensions of 127.76 × 85.48 × 14.22 mm, as shown in Figure 1A, which defines the minimum size for the device development.

In this study, a low-cost but high-performance digital Raspberry Pi camera v2.0 was used to capture images of the microplate. This camera’s electronic board not only has a compact dimension of 32 × 32 mm with an affordable retail price of AUD 25.00, but it also shows adequate sensitivity for optical signals in the visible spectrum. The field of view (FOV) of the camera is an essential feature [19] for the design of the device. The focal length of the Raspberry Pi camera requires the object to be placed further than 50 mm to guarantee the quality of the taken images. In addition, to support the image processing in the later stage, four black reference points were equipped around the microplate, as shown in Figure 1A,C. Therefore, the height of the camera is optimally set at 112 mm for its FOV to cover both the microplate and the reference points, as shown in Figure 1B,C. By placing the camera along the central axis of the microplate, the captured images are symmetric to minimise the optical distortion, or aberration effect, of the circular wells from the centre of the plate to the outer edges. 

#### 2.1.2. The White Light Sources

An essential component for colorimetry measurement is the light source because it provides the optical input to the testing solution. When the white light illuminates the semi-transparent substance, one portion of the light will be reflected from the surface of the substance, another portion will be absorbed by the substance, and the rest of the light will transmit through and escape from the substance, called the transferred light, as shown in Figure S1. From the colorimetry principle, the concentration of chemicals affects the absorption property of the substance, in that, the higher the chemical concentration, the more the light will be absorbed. By using a selective recognising reagent for the targeted chemical, the measurement aims to specifically capture the presence of the targeted chemical among the complex mixture of other chemicals. Researchers can detect the presence of the targeted chemicals in the solution by estimating how much the light intensity varies after transmitting through or reflecting from the colour medium [27]. In this device, a white light source is employed since it can provide a full visible spectrum for the colorimetry measurement. In addition, the light source satisfies other design requirements, i.e., it can provide even illumination over the microplate’s surface to minimise the interference noise from shadows on the microplate, minimising the footprint of the light source to minimise the dimensions of the prototype and adding the flexibility to change light sources for various types of colorimetry measurement that may be used in the future. In this device, four cool white LEDs with a 120-degree beam angle were placed at the corners of the microplate. The white LEDs, purchased online from CH_Town Electronics, have a colour temperature of 4000–4500 K with 500–600 lumen output for the maximum 5 W output power. The LEDs are orientated so that the beam direction is perpendicular to the plate, and the distance from the LED to the plate’s surface is 70 mm. These LEDs can be powered by a 9 V battery or the Raspberry Pi module, increasing the portability of the device. Moreover, the LEDs are detachable for easy replacement with different light sources to support other colorimetric measurements.

#### 2.1.3. Main Hardware Body

To minimise interference from ambient environmental light, all optical components, including a 96-WM, a light source, and a camera, are enclosed within a box. The box is fabricated from polylactic acid (PLA) material using 3D-printing technology, which is affordable and common on the market. All inner components are fixed in position to prevent shaking and blurring of colour images. There are four black squares at each side of the testing plate, to be used as reference points to support the image processing, as shown in Figure 1C.

### 2.2. Reagent Kits and Reaction Mechanism 

The commercial test reagent of glucose and creatinine (Thermo Fisher Scientific, Australia) are used. Both reagents are designed to give quantitative results for glucose and creatinine concentrations in urine samples. Each test provides a set of standards, which can be used to generate a standard fitting curve, showing the relationship between colour intensity and concentration. MATLAB (Version R2021b) was used to perform the image processing to identify the colour intensities of the testing samples, the standard fitting curve and to determine the concentrations of the biomarker in the testing solution. 

#### 2.2.1. Creatinine Test Reagent and Sensing Mechanism 

The creatinine test reagent follows the Jaffe reaction, a specific technique of colorimetry for monitoring creatinine in biofluids. In the Jaffe method, when creatinine reacts with picric acid in an alkaline environment, it develops a red colour [28]. The red intensity varies proportionally depending on the creatinine concentration in the solution. From the set of 7 standards with concentration increasing from 0 to 1.77 mmol/L (20 mg/dL), the operator can produce the standard fitting curve for creatinine. When testing urine samples, the operator will dilute the samples with distilled water to lower the concentration of creatinine to fit in the range of detection. After being mixed with the testing reagent and incubating at the room temperature for 30 min, if creatinine is present in the sample, the mixture will turn to a red colour, which will be matched to the fitting curve to calculate the creatinine concentration. In clinical measurement, the abnormal creatinine concentration in urine samples may be much higher than the detection range; thus, further dilution and re-testing are recommended to achieve a reliable result. (For further detail of the commercial creatinine test kit, please refer to the supporting document).

#### 2.2.2. Glucose Test Reagent and Sensing Mechanism 

The glucose test reagent uses colorimetric substrate, horseradish peroxidase (HRP) and glucose oxidase as the recognising reagents. When combined with any glucose in the urine sample, the mixture turns from colourless to a pink solution, and the strength of the pink colour level corresponds to the concentration of glucose in the urine sample. A beta-D-glucose standard is prepared to give the standard fitting curve with the detection range of 0 to 32 mg/dL of glucose. When testing, the urine sample is diluted with a glucose buffer to a certain level, then mixed with the other dye reagents, i.e., the glucose substrate, the horseradish peroxidase (HRP) and the glucose oxidase. After being mixed with the urine sample and left for incubation at room temperature for 30 min, if glucose is present in the solution, the mixture will turn a pink colour, which will be captured and analysed to give the concentration of glucose in the urine sample. Similarly with the creatinine test kit, if the glucose in the urine samples is over the detection range, further dilution and re-testing are recommended to obtain a reliable and accurate measurement of glucose concentration. (For further detail of the commercial glucose test kit, please refer to the supporting document).

### 2.3. Image Processing Algorithm 

#### 2.3.1. Alignment

To reduce the complexity of image alignment and segmentation, the device includes four reference points, shown in Figure 1C. The points are used to align the test and calibration images, and they are also used as a reference to define the segmentation locations of the test and reference colour blocks in 96-WM. The details on detecting the location of the four reference points and performing alignment can be found here [29].

#### 2.3.2. Calibration

A single test image contains both the test and reference colour blocks in a 96-WM. To measure the correct concentration of a biomarker, it is important that the illumination across the whole image is homogeneous. In practice, this is not possible as the illumination at different points across the image can be affected by variations among the four light sources and light source intensity over time, variations in camera sensitivity within an image regarding the flat fielding effect and colour sensitivity, and variations between devices. To make the biomarker concentration measurement invariant to these changes, a calibration process has been developed that uses an image of a white block to correct the test image, as reported previously [29].

#### 2.3.3. Segmentation and Intensity Measurement

**Figure 2:**
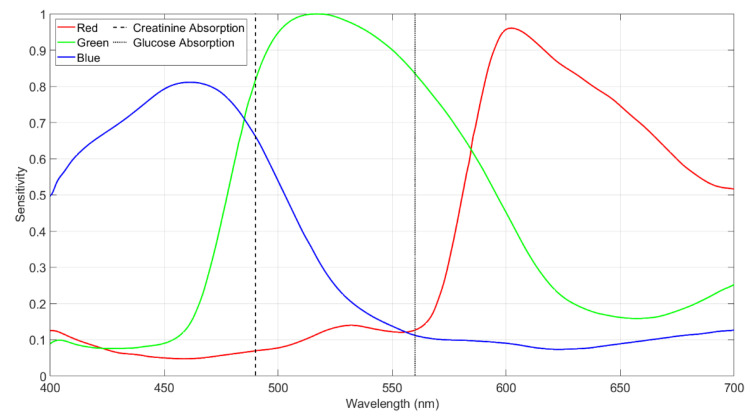
The Raspberry Pi SONY IMX219 camera RGB sensitivity and the absorption wavelength of the creatinine and glucose.

Following the image alignment process, the position of the 96 wells within an image will not change significantly. Therefore, the segmentation locations for each well are defined relative to the four reference points. These segmentation locations are indicated in Figure S2 by red borders. To obtain three single RGB intensity values from each well, intensity values that correspond to the highest frequency of occurrence in the respective RGB histograms are found using the method described in [19,30]. The intensity is calculated in this way to ensure that it is robust to different noise sources that may affect the image, such as sensor noise and artefacts such as reflections, glare, bubbles, and parts of the well that do not include the solution. The Raspberry Pi camera can capture the whole visible spectrum, but to obtain a single intensity value, the three RGB intensity values can be combined in a weighted average based on the camera sensitivity at absorbance wavelengths [19,30]. The absorbance wavelength has been used in previous analytical devices because it is constant and has the largest dynamic range for measurement with respect to the concentration of the analyte [31,32,33]. The peak absorbance wavelength for creatinine is 490 nm, and the peak absorbance wavelength for glucose is 560 nm (Refer to the manual documents of commercial test kit of glucose and creatinine in the supporting information document). The sensitivity of the Raspberry Pi camera in the three RGB channels, from the Sony IMX219 datasheet and [34], is shown in Figure 2, along with the absorption wavelengths. The weighted average of the three RGB components is as follows:

(1) $$y=aI_{R}+bI_{G}+cI_{B}$$

where *I_R_*, *I_G_*, and *I_B_* are the red, green, and blue intensity values in the image and *a*, *b*, and *c* are the coefficients corresponding to the sensitivity of the camera at the peak absorbance wavelength. The *a*, *b*, and *c* coefficients are found using the RGB sensitivities, namely, $R_{S}$, $G_{S}$, and $B_{S}$, respectively, as follows:

(2) $$a=\frac{R_{S}}{R_{S}+G_{s}+B_{S}}$$

(3) $$b=\frac{G_{S}}{R_{S}+G_{s}+B_{S}}$$

(4) $$c=\frac{B_{S}}{R_{S}+G_{s}+B_{S}}$$

The camera sensitivity values for creatinine and glucose are $R_{S}=0.07$, $G_{S}=0.82,$ and $B_{S}=0.66$ and $R_{S}=0.13$, $G_{S}=0.83,$ and $B_{S}=0.11$, respectively, as shown in Figure 2.

#### 2.3.4. Measurement

The absorption of the solution is measured using optical density (OD), which is a logarithmic intensity ratio of the light reaching the solution to the light transmitted though the solution as follows [35]:

(5) $$OD=-{log}_{10}\frac{I_{o}}{I_{t}}$$

where $I_{o}$ and $I_{t}$ are the intensities of the incident and transmitted lights, respectively.

The transmitted light is the intensity at the absorption wavelength found in Equation (5) and the incident light is estimated based on the intensity values of the empty wells. As the intensity of the wells can change slightly from the centre to the edges of the plate, the mean of the empty well intensity in the 5 × 5 neighbourhood of the current well is used to estimate the incident light intensity. To estimate the local incident light intensity, first, a histogram of the intensities of all 96 wells is constructed as follows:

(6) $$H_{y}\left(y_{k}\right)=n_{y_{k}}$$

where k=0,1,…,L−1 and $n_{y_{k}}$ represents the number of times that the intensity level $y_{k}$ appears across the 96 wells. Considering the wells containing the solution will have varying intensity values and empty wells will have similar values, the intensity of the empty wells will be the value that appears most frequently in the histogram. The most frequent empty well intensity value can be determined using a maximum operator as follows:

(7) $$w=n:\left(H_{y}\left(n\right)=argmax\left(H_{y}\right)\right)$$

To find the mean value of the intensity of the empty wells adjacent to the current well, let $S_{y}$ be the set of all well intensities in the 5 × 5 neighbourhood. The set of empty wells, $S_{w}$, is then determined as those with an intensity greater than or equal to 95% of the most frequent empty well value, w, as follows:

(8) $$S_{w}=\left\{S_{y}\left(p\right):S_{y}\left(p\right)\geq 0.95w\right\}$$

where p=1,2,…P and P is the cardinal number of $S_{y}$. 

The mean intensity of the local empty wells is found as follows:

(9) $$w_{L}=\frac{\sum_{q=1}^{Q}S_{y}\left(q\right)}{Q}$$

where q=1,2,…Q and Q is the cardinal number of $S_{w}$.

The *OD* is calculated using the mean of the empty well intensity in the 5 × 5 neighbourhood to estimate the incident light input as follows:

(10) $$OD=-{log}_{10}\frac{w_{L}}{y}$$

To determine the actual concentration from an *OD* value, the line of best fit is found using the standard concentration samples. The known concentration values have a logarithmic relationship to the *OD* values; therefore, the log value of the concentration is used when determining the line of best fit. The diluted concentration value is found as follows:

(11) $$C_{d}=e^{\left(xOD+z\right)}$$

where x and z are the linear coefficients from the line of best fit.

The actual concentration value is then determined by multiplying the diluted concentration value with the dilution factor, $d_{f}$, as follows:

(12) $$C=C_{d}d_{f}$$

### 2.4. Sample Evaluation Procedure 

To evaluate the performance of the device, an evaluation protocol was established with two stages. In stage 1, the device performance was evaluated by comparison with the results of a commercial 96-WM reader instrument. In stage 2, the device performance using the commercial test reagent to detect the biomarker in patient urine samples was evaluated by comparison with clinical values. 

#### 2.4.1. Commercial Comparison

The performance of the prototype device was evaluated by comparing results obtained by the device using commercial test reagents to detect biomarkers in the biofluid samples with results from a commercial microplate reader. A microplate reader is a common instrument used in clinical and research laboratories. It can provide specific wavelength luminescence for different colorimetry measurement. In addition, with the high-sensitive photocell, the reader can examine the colour of the single well on the microplate. By testing the colour wells in an enclosed dark-space with precise illumination and detection, the reader gives an accurate colour value for the individual well. The microplate reader for this evaluation is the multi-mode microplate reader, SpectraMax iD5. When working with the glucose and creatinine commercial kits, the SpectraMax iD5 is required to use the bottom-read luminescence method, thus using the transparent microplate as opposed to the white opaque microplate used in the prototype device. The general steps of obtaining a measurement using the commercial test kits in the 96-WM with the microplate reader and the proposed colorimetry device are described in the flowchart shown in Figure S3.

**Figure 3:**
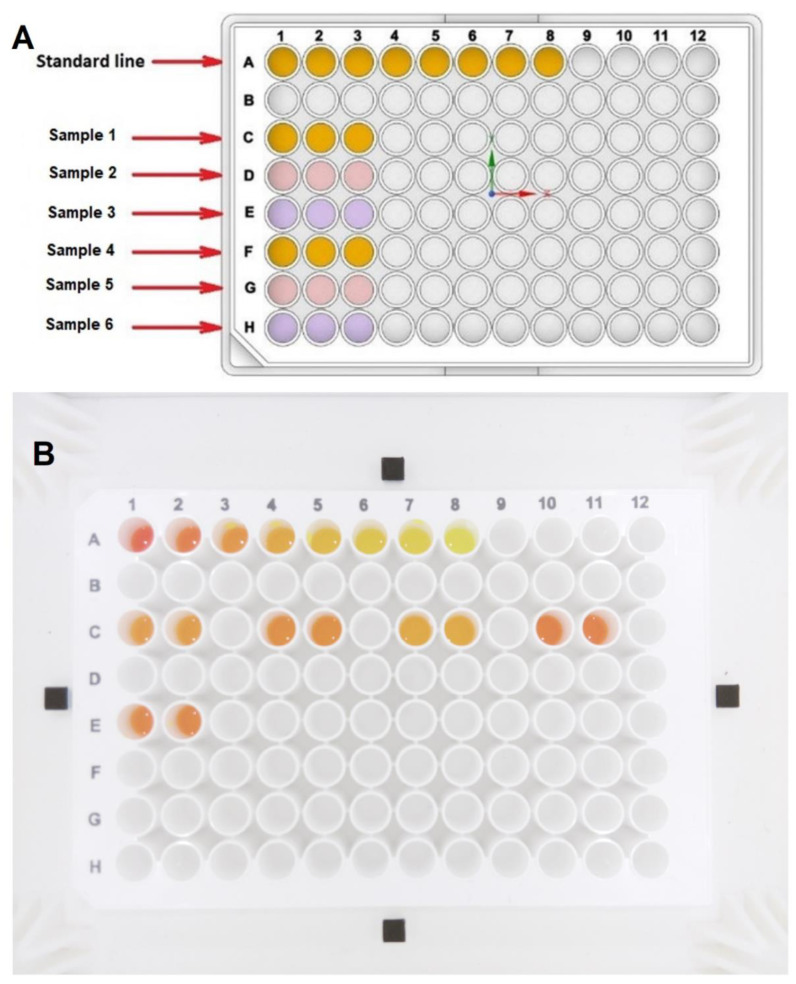
The 96-well microplates with the example of creatinine solutions. (**A**) The sketch of the 96-WM with the standards in wells A (1–8) and the testing solution in other wells; (**B**) An image of the creatinine solutions taken by the Raspberry Pi camera in the proposed colorimetry device.

When preparing the solutions for the microplate test, the standards are often placed in the wells across the top row of the plate, and the test solutions are prepared in the other wells, as shown in Figure 3A. For the measurement with the microplate reader, all chemicals were poured into a transparent plate to support the bottom-reading process. Then, all chemicals were transferred to the white opaque microplate for measurement in the device, as shown in Figure 3B. Finally, the white plate with chemicals was put in the device for image capture prior to colour analysis. An image of the test in the developed device is shown in Figure 3B. The results of testing samples obtained from the colorimetry device and the microplate reader were compared with the expectation that the calculated results from the device and the microplate reader will be close to the real concentration of the biomarkers in the prepared samples.

#### 2.4.2. Clinical Comparison

In the clinical evaluation stage, the performance of the device was evaluated with urine samples collected from patients at Flinders Medical Centre, South Australia, Australia. Following ethics approval, 73 urine samples from renal patients were collected. Due to the scope of the ethical approvement, only creatinine values were compared in this clinical evaluation stage. The urine samples were tested using the device and compared with the clinical values provided by SA Pathology. When using the proposed colorimetry device, the samples were prepared following the standard of procedure from the commercial test reagent manual. The prepared testing solution was then placed in a 96-WM and left in room temperature for a 30-min incubation. Then, the 96-WM filled with the colour testing solutions was placed in the device for imaging and analysis.

## 3. Results and Discussion

### 3.1. Evaluation of the Device in Compared with a Commercial Microplate Reader

**Figure 4:**
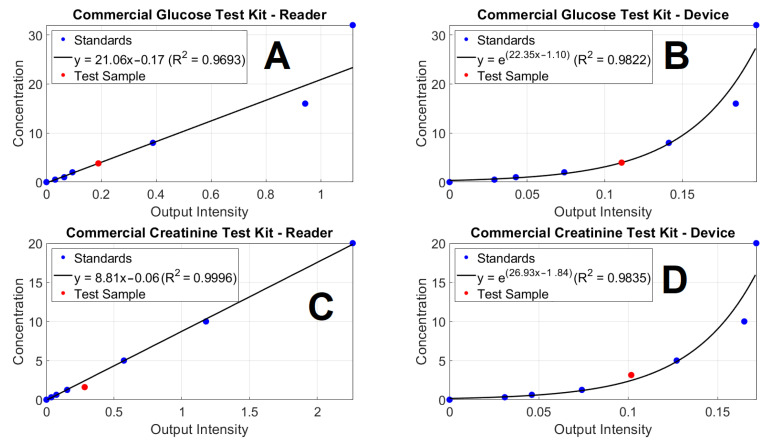
The unknown testing samples (red dot) are illustrated in the graphs of the standards from (**A**,**B**) glucose and (**C**,**D**) creatinine commercial test reagents, operated by (**A**,**C**) a microplate reader and (**B**,**D**) the developed device.

For the comparison of the device with the commercial reader, standard samples were compared. As shown in Figure 4, the device and the commercial reader produce a similar fit for the standard samples. The log of the known concentration values was used with the device for a linear fit. In this comparison, one of the standard samples was used as an unknown concentration; the 4 mg/dL glucose sample and the 0.221 mmol/L (2.5 mg/dL) creatinine sample and the concentration of these samples was determined using the linear fits from Figure 4. Table S1 shows that the device produced a more accurate concentration value for the unknown samples for both the glucose and creatinine measurements. When testing the samples with the known concentrations for glucose and creatinine, the developed device can detect and indicate the biomarkers concentrations, within 10% of the gold standard [16], as shown in Table S1. These results have confirmed the ability of the device to perform as a reader for the measurement of glucose and creatinine using commercial test kits and the 96-WM.

### 3.2. Clinical Evaluation

**Figure 5:**
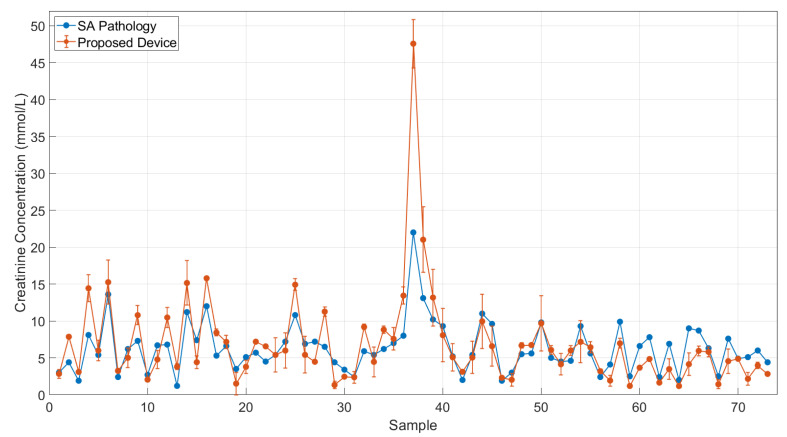
Comparison of creatinine concentrations measured by device and obtained from SA Pathology for 73 urine samples. The biggest variation is in sample 37, which has a strong intensity in the background colour. Standard deviation is for two replicate samples.

The results obtained with the device and from SA Pathology are shown in Figure 5. The device indicates a very consistent response when compared with the clinical values throughout the set of 73 urine samples from kidney-related disease patients. The results were statistically analysed using the paired samples *t*-test that gave *t* = 1.1334 with 72 degrees of freedom and *p* = 0.2695. This indicates there was no significant difference between the creatinine concentrations measured by the proposed device and those provided by SA Pathology. On average, the creatinine concentration measured by the proposed device was 0.52 mmol/L higher (95% Confidence Interval: 0.00, 1.04) than the concentrations provided by SA Pathology.

From the information provided for the creatinine commercial kit (refer to the supporting information document), the sensitivity value based on the mean optical density plus two standard deviations for a zero-concentration standard assayed 20 times is 0.0017 mmol/L. For comparison, a zero-concentration standard was assayed 17 times in the proposed device and returned a sensitivity value of 0.22 mmol/L. This value is higher than that specified by the commercial kit, but still acceptable given that the clinical creatinine values had a range of 1.2 mmol/L to 22 mmol/L.

There are a few samples for which the device indicates higher value than that of the clinical value. One reason for the variation can be deduced from the operating procedure of the commercial test reagent. The creatinine test reagent provides the standards, which can be used to generate a fitting curve in a certain range of detection. The urine test samples are diluted into a certain level, which can be up to 100 times according to the test manual, before being mixed with the testing chemicals. From the colorimetry measuring principle, the purpose of diluting the urine sample is to bring the creatinine concentration of the sample into the detection range of the fitting curve. However, for the device, the fitting curve of the creatinine test kit is an exponential curve, as shown in Figure 4. If a creatinine concentration is in the high range of the fitting curve, a small variation in intensity can result in a large difference in the calculated concentrations. For example, as shown in Figure S4, the two pairs of C1-C2 and C3-C4 have the minor difference in intensity (0.01 unit), but the variation in the concentrations depends on the region of the curve. For C3-C4, the variation in concentration is only 0.58 units, but for C1-C2, the variation is 4.04 units. The difference between these two pairs of examples is because the pair C1-C2 is located in the high range of detection, whilst the pair C3-C4 is located in the medium range of detection. This can explain some of the variations between the device and the clinical values. The solution for this is provided in the protocol of the commercial test reagent. If the urine samples contain a significantly high concentration of creatinine, the 10-time dilution, used to obtain these results, will not be enough, and these samples require further dilution.

Another possible reason for the variation could come from the background colour of the urine samples. Colorimetry is a measuring method working on monitoring the change or the development of the colour from the testing solutions. In this research, the testing solutions are urine samples, which are comprised of multiple compounds and chemicals with complicated structures. If the urine sample has a very strong colour background, such as dark yellow or orange, it may add interference to the colour evaluation. To deal with this issue, the further dilution of the urine sample can be a possible solution, as high dilution can reduce the background colour of the sample.

### 3.3. Dilution Factor on Creatinine Results

To examine the effect of the dilution factor on the creatinine result, a urine sample (Sample 37) was diluted by multiple factors with distilled water, i.e., 10, 50, 100, 150, and 200 times. Two diluted samples with the same dilution factor were then tested with the device. Table 1 shows the original dilution factor of 10 times had a higher standard deviation (SD) when compared to the samples with stronger dilution factors of 50, 100, and 150. However, if the sample is diluted too much (with a dilution factor of 200), then it may fall below the lower limit of detection and the standard deviation will increase. Therefore, increasing the dilution can improve the accuracy of the concentration measurement for higher concentration samples. 

From the evaluations and the results, the device has shown good performance when compared to the clinical values. Having simple instruction, affordable fabrication, and compact size, the device has the potential for use as a portable colorimetry reader for commercial test reagents with a microplate for rural and remote areas.

## 4. Conclusions

In this study, a proposed colorimetry device using commercial test kits and a 96-well microplate to measure urine creatinine was developed and evaluated through clinical tests. The device is designed and fabricated with affordable materials for simple operation with an easily replaceable light source, a low-cost of 25 AUD and a high-performance digital camera for colour image capture, and compact size. The device has shown an equivalent response to the commercial microplate reader for measuring urine creatinine levels, and comparable patient urine creatinine results from a clinical pathological laboratory. The device has the flexibility for measuring multiple chemicals and biomarkers in biofluids. Furthermore, results have confirmed that the calibration method provides results that are robust to variations in light source intensity across the 96 wells. Therefore, the proposed colorimetry device has the potential to work as a portable colorimetry reader, supporting the chemical measurements in remote and rural medical facilities.

## Figures and Tables

**Table 1:** The effect of dilution factor on creatinine results with two samples measured for each dilution factor.

Dilution Factor	1st Sample	2nd Sample	Average	SD of Two Samples’ Results
10	2.83	5.31	4.07	1.75
50	6.00	6.30	6.15	0.21
100	7.14	7.41	7.28	0.19
150	7.28	7.64	7.46	0.25
200	5.28	6.68	6.68	0.99

## Supplementary Materials

Supplementary data to this article can be found online at https://www.mdpi.com/article/10.3390/bios12090723/s1, Figure S1: Possible progress pathways for the light coming to an optical semi-transparent substance; Figure S2: An example of a calibrated test image with red coloured borders indicating the segmentation locations of the 96 wells; Figure S3: The RGB histograms and the detected RGB intensities for well A1 in Figure S2; Figure S4: The RGB histograms and the detected RGB intensities for well A6 in Figure S2; Figure S5: The flowchart of general steps of the measurement using commercial test reagents in the 96-WM with a microplate reader and the proposed colorimetry device; Figure S6: The effect of the exponential fitting curve in calculating the high concentration of creatinine in urine sample; Figure S7: The picture of the real colorimetry device: (A) The outer view; and (B) The inner view with the 96 well microplate; Table S1: The prepared concentrations for glucose and creatinine samples, the results obtained from the SpectraMax iD5 microplate reader and the results obtained from the developed colorimetry device.
