# Supplementary material for: Portable Colorimetric Device with Commercial Microplates for Quantitative Detection of Urine Biomarkers: Design, Development, and Clinical Evaluation
Source: Biosensors (Basel). 2022 Sep 4;12(9):723. doi: 10.3390/bios12090723 (PMC9496577; doi:10.3390/bios12090723)
Supplement: Supplementary file 1 [file biosensors-12-00723-s001.zip › biosensors-1873010-supplementary.pdf]

Article

Portable Colorimetric Device with Commercial Microplates for Quantitative Detection of Urine Biomarkers: Design, Development, and Clinical Evaluation

Anh Tran Tam Pham [1], Damian Tohl [1], Qi Hu [1], Jordan Li [2], Karen J. Reynolds [1] and Youhong Tang [1,*]

[1] Australia-China Joint Research Centre for Personal Health Technologies, Medical Device Research Institute, Flinders University, Tonsley, SA 5042, Australia
[2] Department of Renal Medicine, Flinders Medical Centre, College of Medicine and Public Health, Flinders University, Bedford Park, SA 5042, Australia
* Correspondence: youhong.tang@flinders.edu.au; Tel.: +61-8-82012138

Supporting Information

The details of the commercial test kits are described in detail in the manual documents of the commercial test kits of urine glucose and creatinine, which can be obtained from Thermo Fisher Scientific, Australia websites:

For the Invitrogen™ Creatinine Urinary Detection Kit (EIACUN): <https://www.thermofisher.com/order/catalog/product/EIACUN>

For the Invitrogen™ Glucose Colorimetric Detection Kit (EIAGLUC): <https://www.thermofisher.com/order/catalog/product/EIAGLUC>

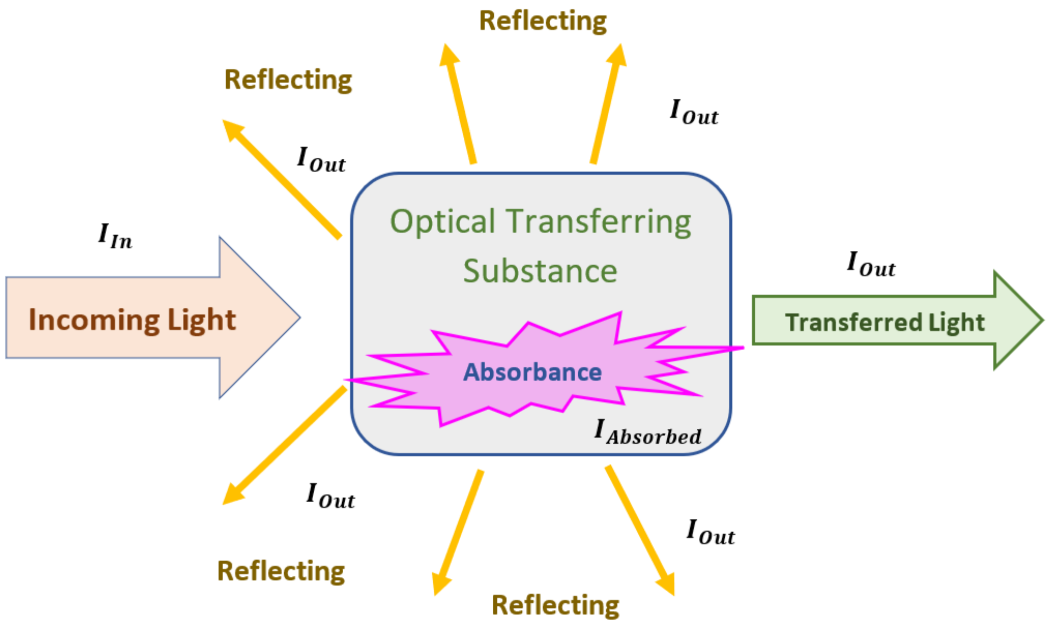

Figure S1. Possible progress pathways for the light coming to an optical semi-transparent substance.

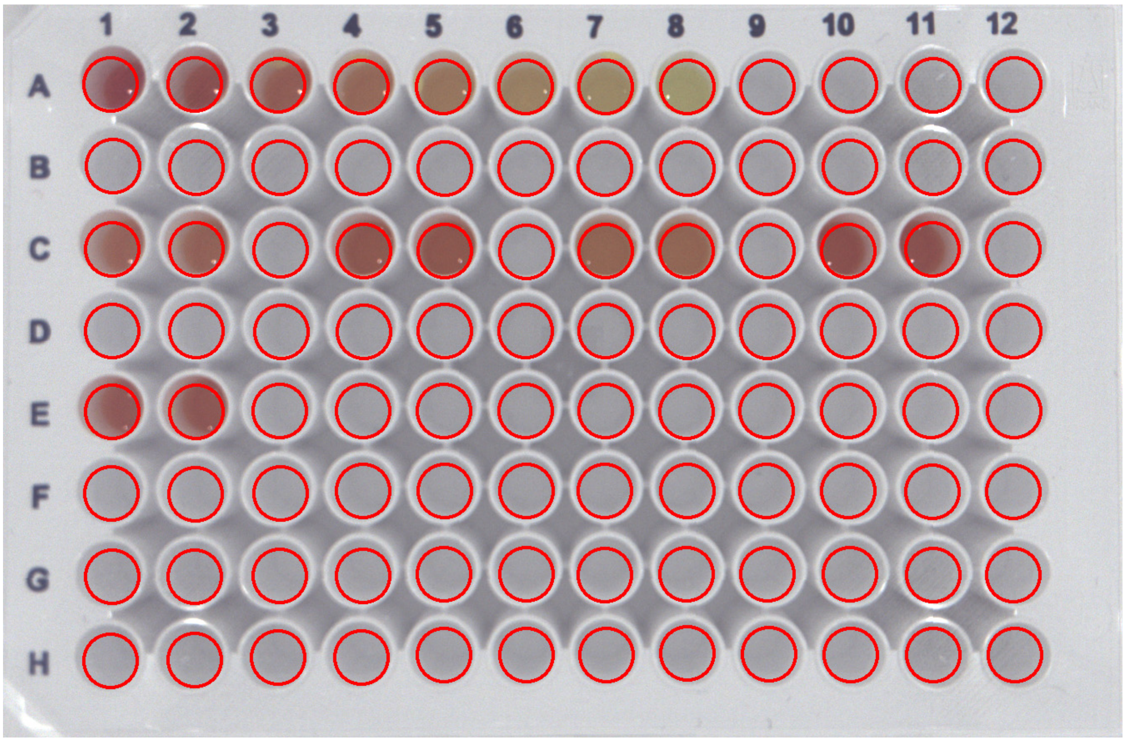

Figure S2. An example of a calibrated test image with red coloured borders indicating the segmentation locations of the 96 wells.

The segmentation locations for the 96 wells in the test plate are shown in Fig. S2. The RGB intensity detection process is robust to different noise sources which may affect the image, such as sensor noise and artefacts like reflections, glare, bubbles and parts of the well which do not include the solution. Fig. S3 shows the RGB histograms and the detected RGB intensities for well A1 in Fig. 1, the sides of the well in the segmentation region correspond to the upper tail in the histogram, which does not affect the detection of the RGB intensities. For comparison, Fig. S4 shows the RGB histograms and the detected RGB intensities for well A6 in Fig. 1, the upper tail in the histogram is reduced because there is less of the well background included in the segmentation region. Therefore, the size of the segmentation region was defined to include a larger area of the solution, as the intensity measurement is unaffected by the sides of the well included in the segmentation region.

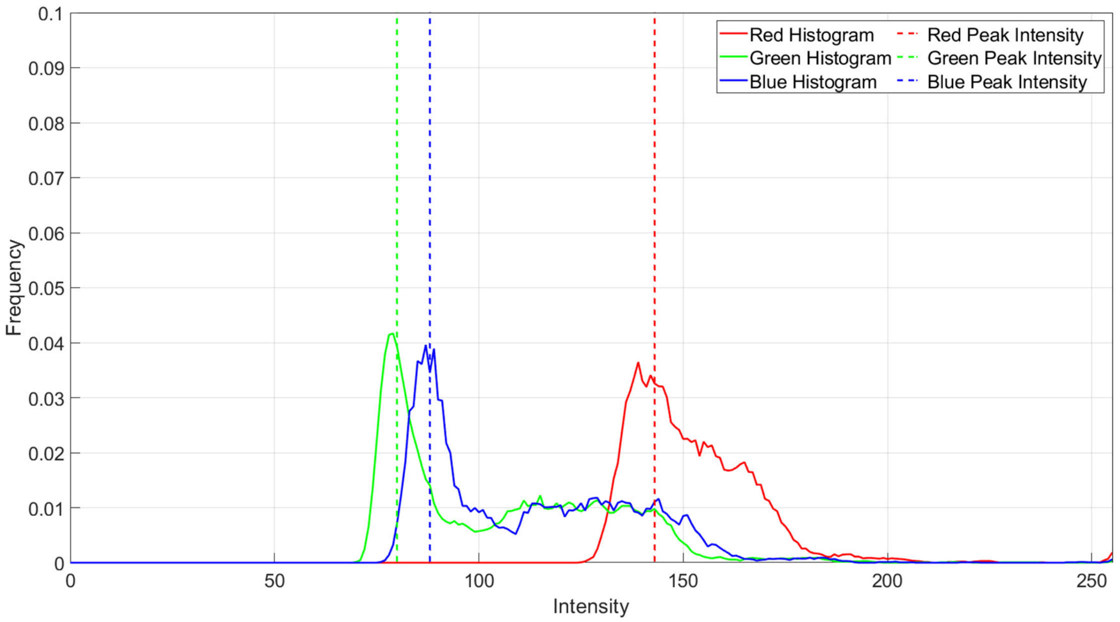

Figure S3. The RGB histograms and the detected RGB intensities for well A1 in Figure S2.

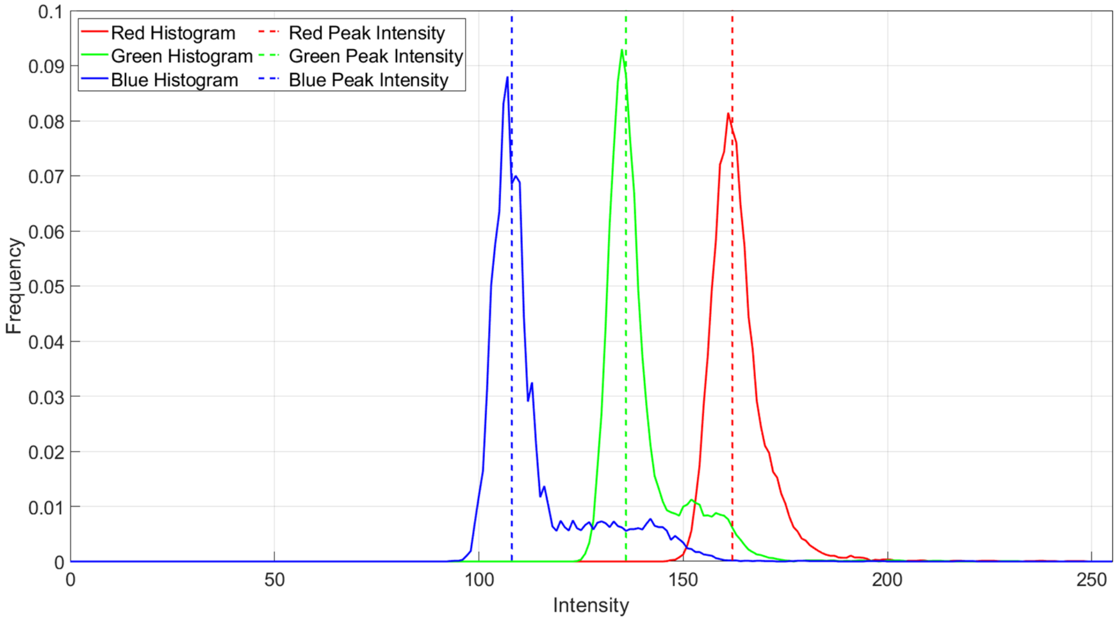

Figure S4. The RGB histograms and the detected RGB intensities for well A6 in Figure S2.

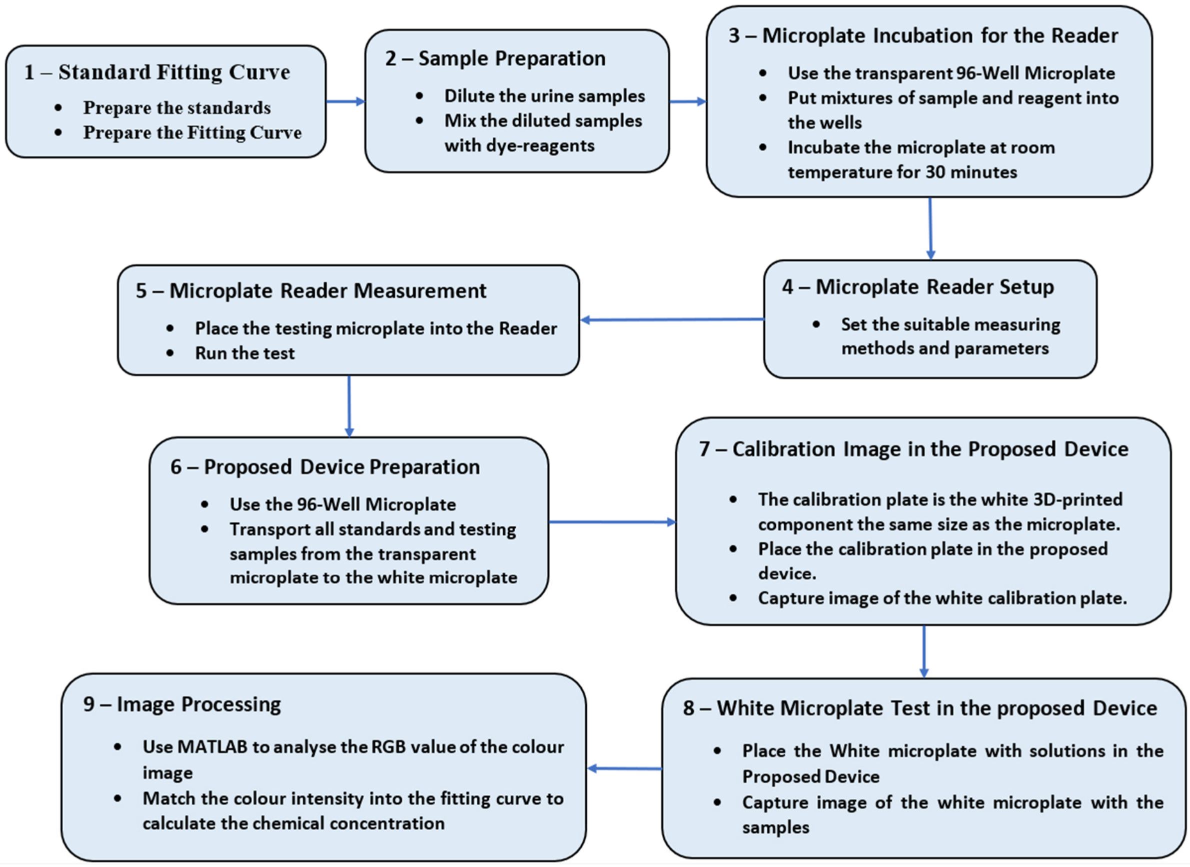

Figure S5. The flowchart of general steps of the measurement using commercial test reagents in the 96-WM with a microplate reader and the proposed colorimetry device.

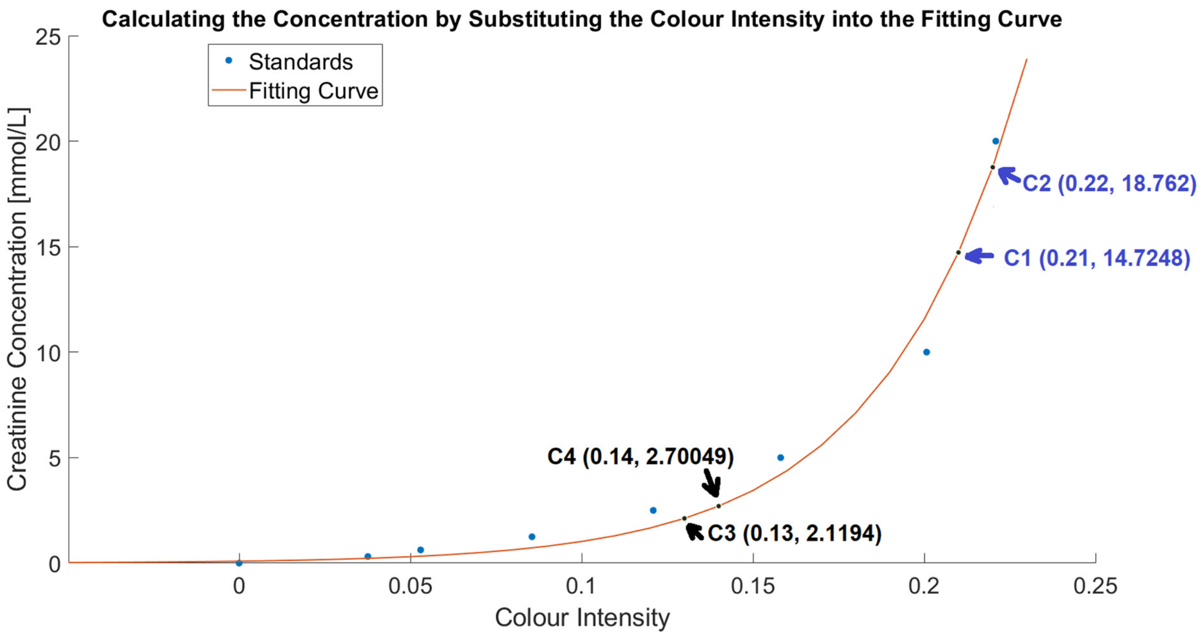

Figure S6. The effect of the exponential fitting curve in calculating the high concentration of creatinine in urine sample. Two data pairs (C1-C2 and C3-C4) have

0.01 unit different in intensities, however, the variations in concentration are 4.0372 and 0.581 units respectively due to the high-range of concentration in the pair C1-C2.

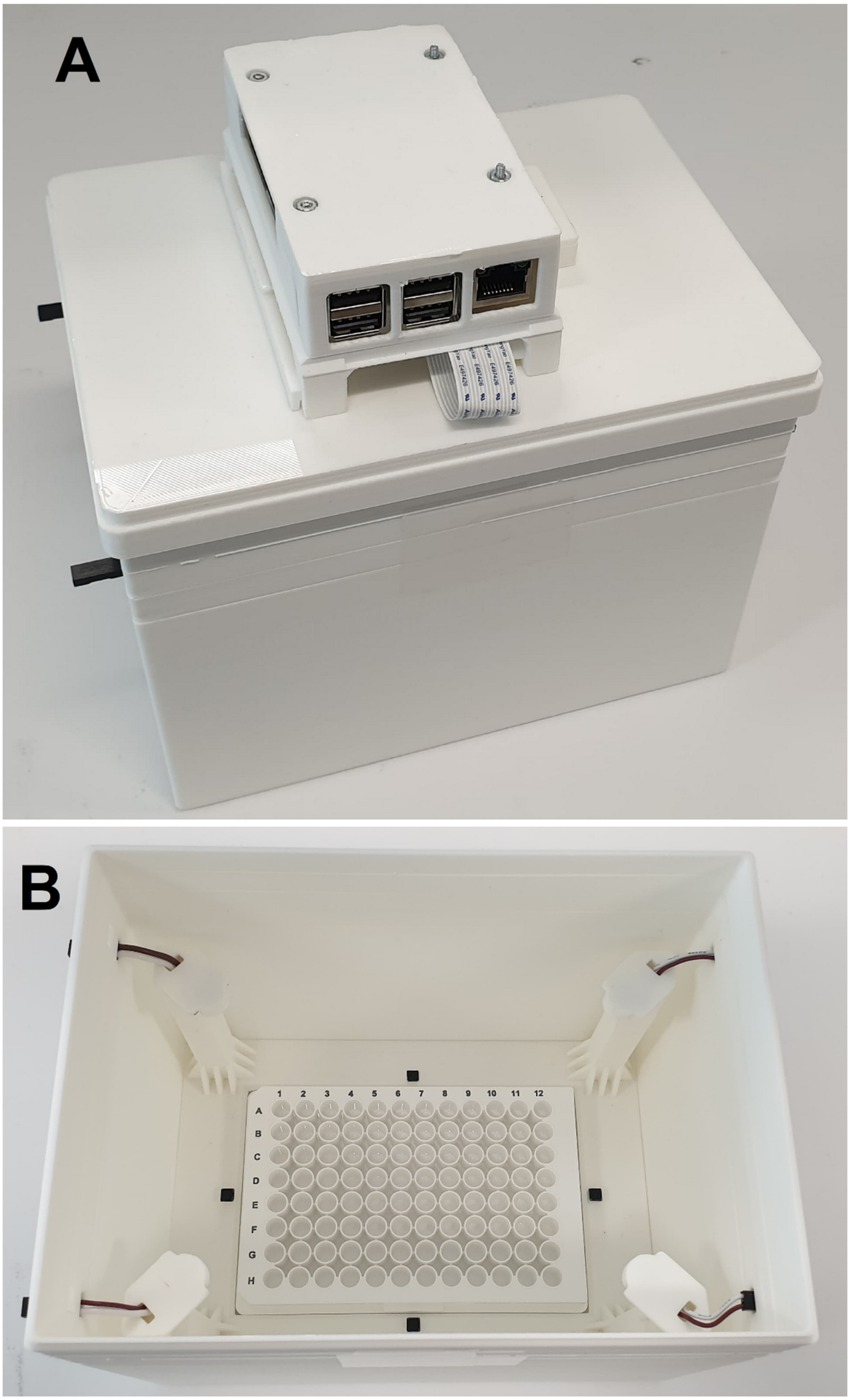

Figure S7. The picture of the real colorimetry device: (**A**) The outer view; and (**B**) The inner view with the 96 well microplate.

Table S1: The prepared concentrations for glucose and creatinine samples, the results obtained from the SpectraMax iD5 microplate reader and the results obtained from the developed colorimetry device.

Biomarker	Prepared Concentration	Result from the Microplate Reader	Result from the Colorimetry Device
**Glucose (mg/dL)**	4.00	3.82	3.97
**Creatinine (mg/dL)**	2.50	2.42	2.46
